# A pairwise approach to revitalize β-lactams for the treatment of TB

**DOI:** 10.1128/aac.00034-24

**Published:** 2024-05-01

**Authors:** Dereje A. Negatu, Wassihun Wedajo Aragaw, Véronique Dartois, Thomas Dick

**Affiliations:** 1 Center for Discovery and Innovation, Hackensack Meridian Health, Nutley, New Jersey, USA; 2 Center for Innovative Drug Development and Therapeutic Trials for Africa (CDT-Africa), Addis Ababa University, Addis Ababa, Ethiopia; 3 Department of Medical Sciences, Hackensack Meridian School of Medicine, Nutley, New Jersey, USA; 4 Department of Microbiology and Immunology, Georgetown University, Washington, DC, USA; Bill & Melinda Gates Medical Research Institute, Cambridge, Massachusetts, USA

**Keywords:** *Mycobacterium tuberculosis*, β-lactam, potentiation, tebipenem, sulopenem, amoxicillin, cefuroxime

## Abstract

The dual β-lactam approach has been successfully applied to overcome target redundancy in nontuberculous mycobacteria. Surprisingly, this approach has not been leveraged for *Mycobacterium tuberculosis,* despite the high conservation of peptidoglycan synthesis. Through a comprehensive screen of oral β-lactam pairs, we have discovered that cefuroxime strongly potentiates the bactericidal activity of tebipenem and sulopenem—advanced clinical candidates—and amoxicillin, at concentrations achieved clinically. β-lactam pairs thus have the potential to reduce TB treatment duration.

## INTRODUCTION

β-lactams are the preferred agents for the treatment of a wide range of bacterial infections and are among the oldest antibiotics in medical practice. They have excellent safety profiles upon long-term use, and their pharmacokinetic and pharmacodynamic properties are well understood (1). They are bactericidal around their minimum inhibitory concentration (MIC) against Gram-positive and Gram-negative pathogens (2) and against *Mycobacterium tuberculosis* (Mtb) (3, 4), a desirable but uncommon attribute of antibiotics. They induce rapid cytolysis of single Mtb cells, as demonstrated for faropenem by quantitative time-lapse microscopy and microfluidics (5). Meropenem is bactericidal to non-replicating drug-tolerant Mtb, where it is thought to target peptidoglycan remodeling that occurs in the non-replicating state (5, 6). In Gram-positive and Gram-negative pathogens, horizontal transfer of β-lactamase genes is the major source of clinical resistance generally associated with high resistance levels (7, 8). However, the emergence of drug resistance in Mtb only occurs *via* genomic mutations (9). Given the functional redundancy of penicillin-binding proteins (PBPs) and peptidoglycan synthesis enzymes (10, 11), canonical target-based mutations conferring phenotypic resistance are uncommon, another favorable property of the β-lactam class. Indeed, numerous indirect mechanisms conferring low-level resistance have been uncovered through *in vitro* mutant selection studies (12, 13), while mutations in direct targets were notably rare, such as *ponA1* mutations resistant to ceftazidime-avibactam leading to moderate resistance (12, 13).

Several screens of β-lactam/β-lactamase inhibitor pairs against Mtb have converged toward meropenem-clavulanate as a promising combination *in vitro* (6), potentiated by amoxicillin (14). However, meropenem-clavulanate given to drug-resistant patients achieved a marginal improvement over non-β-lactam containing regimens (not statistically significant) in clinical trials (15, 16). Although considered a viable alternative for patients with limited therapeutic options, the role of meropenem in programmatic TB treatment has been deemed minimal given the dosing regimen of three times daily intravenous infusions of 1 g each to deliver modest efficacy (17). The high MIC and wide MIC distributions (18) are likely the root cause of the low probability of target attainment and limited clinical utility.

*De novo* discovery of more potent, oral β-lactams to treat TB appears as a promising strategy given the vast collections of β-lactams available in the archives of pharmaceutical companies (19) but is inherently slow and complicated by the chemical instability (20) and poor pharmacokinetics (19) of β-lactams in preclinical species. For rapid bench-to-bedside translation, exploiting candidates in clinical development may constitute a much-needed short-term solution. Indeed, tebipenem-clavulanate recently emerged as a superior, most bactericidal oral carbapenem *in vitro* (21–24). Whether adequate tebipenem concentrations can be achieved clinically remains to be determined (21).

To preempt this limitation, we have considered a one-two punch approach that emerged a few years ago to treat lung infections caused by *M. abscessus* and more recently against the *M. avium* complex: dual β-lactams to overcome target redundancy (25–27) and achieve synergy and potency in line with achievable concentrations in patients. Surprisingly, this approach has not been leveraged yet for Mtb, despite the high level of conservation of peptidoglycan synthesis enzymes in *M. abscessus* and Mtb (10). Furthermore, in *M. abscessus* and stationary phase non-replicating Mtb, 3,3 cross-links predominate and are achieved by L,D transpeptidases (11, 28). Consistent with this unique trait of mycobacteria, both species harbor five homologous L,D transpeptidases (10) thought to be partially redundant.

Inspired by the *M. abscessus* approach, we screened a collection of oral β-lactams belonging to the three major subclasses (penicillins, cephalosporins, and penems, which include carbapenems), either FDA approved or in late clinical development, to identify synergistic pairs that achieve strong bactericidal activity at lower concentrations than their single component’s MIC. Our objectives were to confirm the superior biological profile of tebipenem-clavulanate and to identify potent bactericidal pairs that position dual β-lactams as a promising strategy to shorten TB treatment.

**Fig 1:**
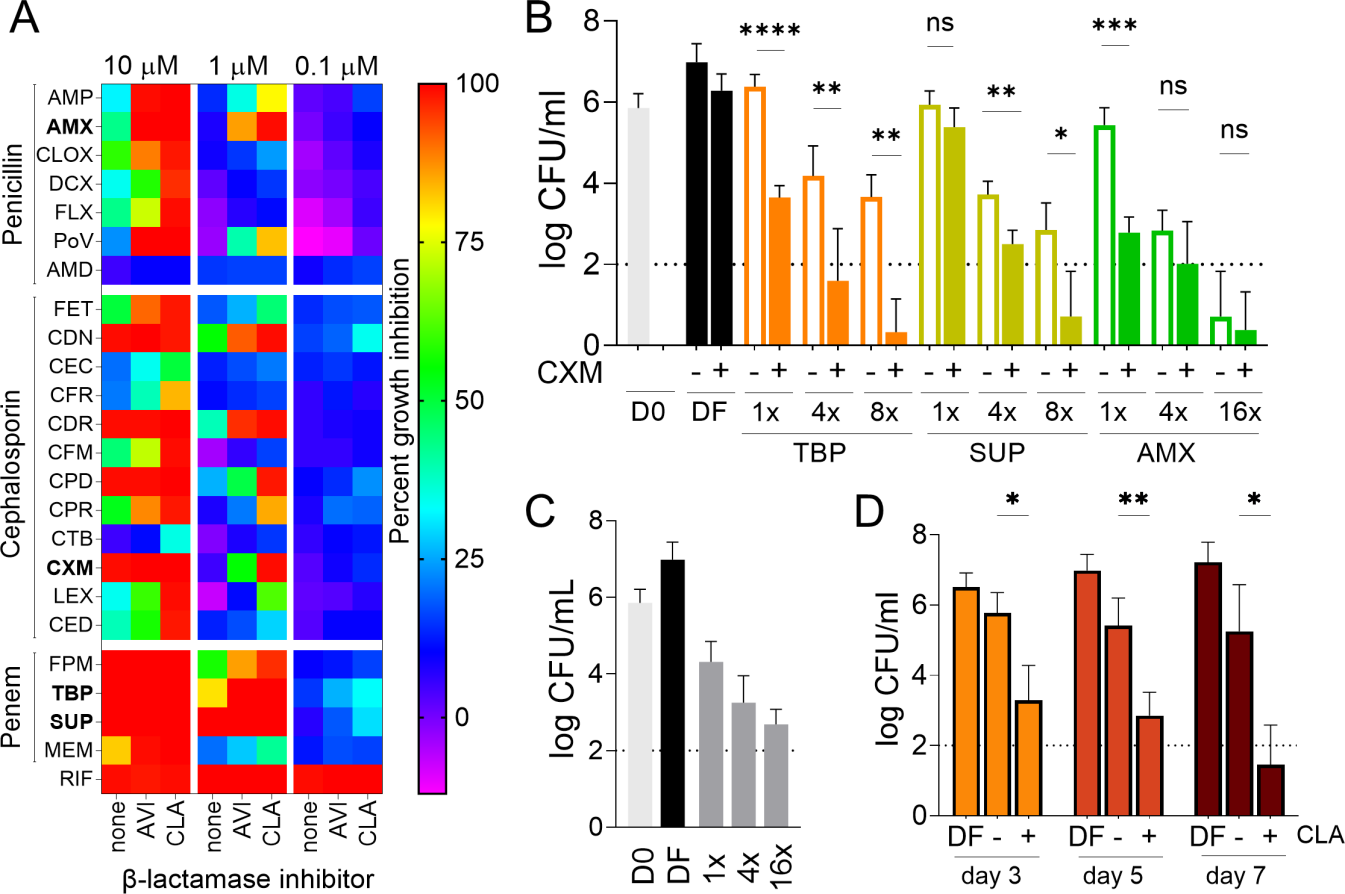
Growth inhibition and bactericidal activity of single and dual β-lactams. (**A**) Growth inhibition of *M. tuberculosis* H37Rv reference strain by oral β-lactams at 10, 1, and 0.1 µM, with and without β-lactamase inhibitor avibactam (AVI, 4 µg/mL) or clavulanate (CLA, 2.5 µg/mL) in 96-well plates. The plates were incubated at 37°C for 7 days at 110 rpm. Growth was monitored by absorbance at 600 nm, and % inhibition was calculated relative to the untreated controls. Tebipenem (TBP), sulopenem (SUP), amoxicillin (AMX), and cefuroxime (CXM) (in bold) were selected for subsequent experiments. Rifampicin (RIF) is included as assay control, and the injectable meropenem (MEM) as a comparator. A complete list of drug abbreviations is provided in Table S1. (**B**) Dose-response bactericidal activity of TBP, SUP, and AMX as single β-lactams (with CLA 2.5 µg/mL) or combined with cefuroxime at 1 µg/mL against Mtb H37Rv, showing bactericidal potentiation when cefuroxime is added to each single agent. Cultures were treated for 5 days with TBP and SUP at 1×, 4×, and 8× their respective MIC in combination with CXM, or 1×, 4×, and 16× the combination MIC for AMX, as indicated. Colony forming units (CFU) were enumerated by plating on Middlebrook 7H11 agar medium. The horizontal dotted lines indicate the lower limit of detection or 100 CFU. (**C**) Bactericidal activity of MEM-CLA at MIC multiples after 5 days of incubation. (**D**) Time-kill experiment showing the impact of CLA on the bactericidal activity of SUP at its MIC (0.5 µg/mL). All experiments were carried out three times independently and the mean/SD (error bars) of the combined results are shown. Data were analyzed by two-way ANOVA with Tukey’s multiple comparisons test. Relevant comparisons are shown; ns: not significant; *P < 0.05, **P < 0.01, *** *P* < 0.001, **** *P* < 0.0001.

First, we carried out a comprehensive three-point screen of commercially available oral penicillins, cephalosporins, and penems (Table S1) at 10, 1, and 0.1 µM against Mtb H37Rv. Each β-lactam was tested in the absence of a β-lactamase inhibitor, with avibactam 4 µg/mL or clavulanate 2.5 µg/mL to reflect average concentrations achieved clinically and follow official guidelines (29, 30). All growth inhibition experiments were performed according to the EUCAST broth microdilution reference method (31) with minor modifications as follows: Mtb H37Rv was inoculated at a starting density of OD_600nm_ of 0.005 in complete Middlebrook 7H9 broth and 96-well plates in the presence of drugs for 7 days at 37°C and 110 rpm with OD_600nm_ as readout. Clavulanate consistently emerged as a more effective β-lactamase inhibitor than avibactam against Mtb (Fig. 1A). Sulopenem was the most potent of all agents tested and its activity appeared independent of β-lactamase inhibitor at these concentrations. The results also confirmed the high potency of the tebipenem-clavulanate combination. Both sulopenem and tebipenem are in phase 3 clinical development (32, 33). Several cephalosporins and amoxicillin achieved complete growth inhibition at 1 µM in the presence of clavulanate. In single-drug growth inhibition experiments, the MIC of tebipenem, sulopenem, amoxicillin, and cefuroxime against Mtb H37Rv were 0.4, 0.2, 0.8, and 0.8 µg/mL, respectively, in the presence of 2.5 µg/mL clavulanate (Table 1).

**TABLE 1:** Growth inhibitory activity (MIC_90_, or concentrations that inhibit 90% of bacterial growth in μg/mL) of single and dual β-lactams against a panel of *M. tuberculosis* clinical isolates^*a*^

Mtb strain^*b*^	CXM-CLA^*c*^	TBP				SUP				AMX		MEM-CLA	RIF
		Alone	+ CLA	+ CLA CXM^*d*^	+CLA-AMX	Alone	+ CLA	+ CLA CXM^*d*^	+CLA-AMX	+CLA	+ CLA CXM^*d*^		
H37Rv (lineage 4)	0.8	2.0	0.4	0.03	0.1	0.4	0.20	0.06	0.10	0.8	0.2	1.5	0.0020
Mtb L1.2.1	0.5	3.0	0.4	0.06	0.2	0.8	0.40	0.10	0.20	0.8	0.2	1.0	0.0025
L2 (ITM 500945)	1	5.0	0.5	0.03	0.4	0.8	0.25	0.06	0.20	2.5	0.5	1.5	0.0030
L3 (ITM 500949)	0.5	3.0	0.4	0.02	0.3	0.8	0.25	0.03	0.13	1.5	0.1	1.5	0.0030
L4 (ITM 500952)	0.5	2.5	0.4	0.06	0.4	0.8	0.40	0.06	0.20	1.5	0.1	1.5	0.0025
L5 (ITM 500955)	6.0	8.0	1.5	0.50	0.8	2.0	1.00	0.50	2.00	16.0	8.0	3.0	0.0120
MDR R279 (lineage 2)	0.8	1.5	0.4	0.06	0.2	0.2	0.10	0.02	0.10	2.0	0.1	1.5	>32
MDR AT787 (lineage 2)	2	4.0	0.8	0.08	0.8	0.5	0.25	0.12	0.20	6.0	2.0	3.0	>32
MDR R543 (lineage 2)	0.5	3.0	0.4	0.06	0.3	0.8	0.20	0.10	0.20	1.5	0.5	1.5	>32

^*a*^ The concentration ranges were 0.008 to 8 μg/mL for tebipenem (TBP), sulopenem (SUP), and meropenem (MEM), and 0.02 to 16 μg/mL for 316 amoxicillin (AMX) and cefuroxime (CXM). Rifampciin concentration ranges were 0.002 to 2 μg/mL against susceptible strains and 0.03 to 32 317 μg/mL against resistant strains.

^*b*^ Mtb L1.2.1 (lineage 1) was kindly provided by Dr Jeremy Rock (34); BCCM/ITM accession numbers are provided for lineages L2 to L5 (35); MDR (multidrug resistant) strains were provided by Dr Robin M Warren and are resistant to isoniazid and rifampicin (RIF) (36, 37).

^*c*^ CLA added at a fixed concentration of 2.5 μg/mL.

^*d*^ CXM added at a fixed concentration of 0.25 μg/mL.

In other mycobacteria, combining two β-lactams of different subclasses leads to potentiation and is thought to overcome target redundancy. These observations, together with the results shown in Fig. 1A and published clinical breakpoints, prompted us to select amoxicillin and cefuroxime as the penicillin and cephalosporin that could potentiate tebipenem and sulopenem, the main penem anchors, in pairwise combinations. We also included the amoxicillin-cefuroxime pair, a fully FDA-approved combination. Based on similar investigations in *M. abscessus* (38), we reasoned that additivity in growth inhibition may translate into pronounced bactericidal potentiation. Systematic measurements of dose-response MIC were obtained for single agents and β-lactam pairs against Mtb H37Rv as described above, showing additivity (Table S2). Cefuroxime appeared as the preferred potentiator and was selected for dose-response kill experiments. We quantified the bactericidal activity of tebipenem, sulopenem, and amoxicillin with and without cefuroxime at 1 µg/mL. Clavulanate was systematically added at 2.5 µg/mL as the β-lactamase inhibitor. Exponentially growing cultures of Mtb H37Rv were diluted to achieve a final OD_600_ of 0.005 in Middlebrook 7H9 medium and exposed to single β-lactams in 96-well plates at 1×, 4×, and 16× their respective MIC for 1, 3, 5, and 7 days at 37°C and 110 rpm as previously described (39). Dual combinations of tebipenem or sulopenem with cefuroxime were tested at 1×, 4×, and 8× their combination MIC, and amoxicillin-cefuroxime at 1×, 4×, and 16× amoxicillin’s combination MIC. CFU were enumerated after 20 days of incubation on 7H11 agar supplemented with 0.4% activated charcoal. At 1 µg/mL, cefuroxime alone showed no bactericidal activity (Fig. S1) but significantly potentiated the killing achieved by tebipenem and sulopenem at all concentrations tested (Fig. 1B). At 0.25 µg/mL, tebipenem or sulopenem combined with cefuroxime reduced the initial bacterial burden by 3 to 4 orders of magnitude and killed more than 5 log CFU at 0.5 µg/mL. While official clinical breakpoints are not available for these clinical candidates, 0.25 µg/mL is achieved for at least half of the dosing interval in subjects receiving Phase 3 dosing regimens (32, 40–42). Cefuroxime also potentiated the bactericidal activity of amoxicillin, reducing the initial bacterial burden by almost 4-log at 1 µg/mL, its clinical breakpoint for other pulmonary infections (https://www.eucast.org/clinical_breakpoints) and by 5-log at 4 µg/mL (Fig. 1B). In comparison, meropenem-clavulanate reduced the bacterial burden by 2 log at its MIC of 1 µg/mL (Fig. 1C) and requires three times daily intravenous infusions.

To determine whether growth inhibitory concentrations hold true against representative multidrug-resistant strains and the major Mtb lineages responsible for TB disease around the world (35), we measured the MIC of the pairs shown in Fig. 1B against a panel of clinical isolates (Table 1). We found similar potencies of single agents and pairs against all strains except the L5 representative, which was consistently more resistant to all agents (Table 1). L5 (historically *M. africanum*) exhibits within-lineage genomic diversity and differing gene content compared to H37Rv (43). It is primarily restricted to West Africa, where L5 and L6 together cause up to 40% of human TB (44). Thus, drug susceptibility testing may be required prior to initiating therapy in these regions. The activity of single and dual oral β-lactams against intracellular *M. tuberculosis* remains to be measured to confirm adequate growth inhibition at clinically achievable concentrations, which is a limitation of this study.

As suggested by the results of the initial screen (Fig. 1A) and the dose-response MIC against the strain panel (Table 1), sulopenem was less dependent on clavulanate than tebipenem and amoxicillin for growth inhibition. To determine whether this applies to sulopenem’s bactericidal activity, we repeated the concentration-kill experiment without clavulanate and found it is required to enable sulopenem’s bactericidal activity around its MIC (Fig. 1D).

Treatment shortening is the major objective of TB drug development. Recently, the three to four drug regimen bedaquiline-pretomanid-linezolid (BPaL) without or with moxifloxacin (BPaLM) reduced the treatment duration of multidrug-resistant or extensively drug-resistant (XDR) TB from 18-24 months to 6–9 months (45, 46). However, linezolid is largely bacteriostatic and is discontinued temporarily or permanently in a significant fraction of patients due to mitochondrial toxicity (47), and XDR-TB patients are resistant to moxifloxacin. Based on the strong bactericidal potentiation uncovered in this study, substituting linezolid and moxifloxacin for dual β-lactams in BPaL or BPaLM has the potential to further reduce treatment duration. Tebipenem and sulopenem are in phase 3 against urinary tract infections and could be considered in future TB clinical trials. Oral amoxicillin-cefuroxime could replace meropenem infusions as salvage therapy in XDR-TB patients with limited therapeutic options.

## SUPPLEMENTAL MATERIAL

Supplemental material aac.00034-24-s0001.pdf Tables S1 and S2; Figure S1.

ASM does not own the copyrights to Supplemental Material that may be linked to, or accessed through, an article. The authors have granted ASM a non-exclusive, world-wide license to publish the Supplemental Material files. Please contact the corresponding author directly for reuse.
