# Supplementary material for: A pairwise approach to revitalize β-lactams for the treatment of TB
Source: Antimicrob Agents Chemother. 2024 May 1;68(6):e00034-24. doi: 10.1128/aac.00034-24 (PMC11620507; doi:10.1128/aac.00034-24)
Supplement: Supplemental material — Tables S1 and S2; Figure S1. [file aac.00034-24-s0001.pdf]

# A pairwise approach to revitalize β-lactams for the treatment of TB

Dereje A. Negatu [a,b], Wassihun W. Aragaw [a], Véronique Dartois [a,c,#], and Thomas Dick [a,c,d]

[a] Center for Discovery and Innovation, Hackensack Meridian Health, Nutley, New Jersey, USA

[b] Center for Innovative Drug Development and Therapeutic Trials for Africa (CDT-Africa), Addis Ababa University, Addis Ababa, Ethiopia

[c] Department of Medical Sciences, Hackensack Meridian School of Medicine, Nutley, New Jersey, USA

[d] Department of Microbiology and Immunology, Georgetown University, Washington, DC, USA

Running Title: Oral β-lactams for TB

Keywords: *Mycobacterium tuberculosis*, β-lactam, potentiation, tebipenem, sulopenem, amoxicillin, cefuroxime,

# Corresponding author: veronique.dartois@hmh-cdi.org

**Supplemental Table 1**. Selected features of oral β-lactams and β-lactamase inhibitors used in this study.

| Class | Drug | 3-letter | Generation | FDA status | MW | 10 μM in μg/mL | Commercial source (Catalog#) [a] |
|---|---|---|---|---|---|---|---|
| penicillin | ampicillin | AMP | | Approved | 349.4 | 3.49 | MedChemExpress (HY-B0522) |
| | amoxicillin | AMX | | approved (NCT02381470 for TB) | 419.45 | 4.19 | Sigma-Aldrich (1031503) |
| | cloxacillin | CLOX | | Approved | 475.88 | 4.76 | Sigma-Aldrich (PHR1922) |
| | dicloxacin | DCX | Second | Approved | 492.31 | 4.92 | Sigma-Aldrich (46182) |
| | flucloxacillin | FLX | | Approved | 475.85 | 4.76 | Sigma-Aldrich (SML1023) |
| | penicillin V | PoV | First | Approved | 388.48 | 3.88 | Sigma-Aldrich (PHR2644) |
| | amdinocillin | AMD | First | Approved | 325.43 | 3.25 | MuseChem (32887-01-7) |
| cephalosporin | cefetamet | FET | Third | Phase 4 (NCT04664803) | 548.03 | 5.48 | MedChemExpress (HY-B1894A) |
| | cefditoren | CDN | Third | Approved | 528.56 | 5.29 | MedChemExpress (HY-17452) |
| | cefaclor | CEC | Second | Approved | 385.82 | 3.86 | Sigma-Aldrich (PHR1283) |
| | cefadroxil | CFR | First | approved (NCT02381470 for TB) | 381.4 | 3.81 | Sigma-Aldrich (C0650000) |
| | cefdinir | CDR | Third | Approved | 395.41 | 3.95 | Sigma-Aldrich (C7118) |
| | cefixime | CFM | Third | Approved | 453.45 | 4.53 | Sigma-Aldrich (CDS021590) |
| | cefpodoxime | CPD | Third | Approved | 427.46 | 4.27 | Sigma-Aldrich (32344) |
| | cefprozil | CPR | Second | Approved | 389.43 | 3.89 | Sigma-Aldrich (Y0001371) |
| | ceftibuten | CTB | Third | Approved | 410.42 | 4.10 | Sigma-Aldrich (SML0037) |
| | cefuroxime | CXM | Second | Approved | 446.37 | 4.46 | Sigma-Aldrich (C4417) |
| | cephalexin | LEX | First | Approved | 365.40 | 3.65 | Sigma-Aldrich (PHR1848) |
| | cefradine | CED | First | Approved | 349.4 | 3.49 | Sigma-Aldrich (C0690000) |
| Penem/ carbapenem | tebipenem | TBP | | approved in Japan for pediatric use; Phase 3 (NCT02381470) | 383.49 | 3.83 | MuseChem (161715-21-5) |
| | sulopenem | SUP | | Phase 3 (NCT05584657) | 349.45 | 3.49 | Sigma-Aldrich (PZ0042) |
| | faropenem | FPM | | Approved in Japan and India (NCT02381470 for TB) | 307.3 | 3.07 | Sigma-Aldrich (F8182) |

| | | | | | | |
|---|---|---|---|---|---|---|
| | Meropenem [b] | MEM | Approved | 437.51 | 4.37 | Sigma-Aldrich (M2574) |
| β-lactamase inhibitor | Avibactam | AVI | Approved for parenteral injection; oral prodrug in Phase 1 | 287.23 | 2.87 | MedChemExpress (HY-14879A) |
| | Clavulanic acid | CLA | Approved | 205.09 | 2.05 | MedChemExpress (HY-A0256B) |

[a] Stock solutions of all drugs were prepared in dimethyl sulfoxide (DMSO) except ampicillin, cefaclor and cephalexin which were dissolved in water.

[b] Parenteral agent, included as control since it is used for the treatment of multidrug resistant TB

**Supplemental Table 2**. Growth inhibition checkerboard analysis of selected β-lactams against Mtb H37Rv

| Drug A | Drug B | $MIC_{90}$ (μg/mL) [a] | | FICI [b] |
|---|---|---|---|---|
| | | alone | In combination | |
| Tebipenem | | 0.25 | 0.06 | |
| | Amoxicillin | 0.25 | 0.13 | 0.75 |
| Tebipenem | | 0.25 | 0.06 | |
| | Cefuroxime | 0.50 | 0.13 | 0.5 |
| Sulopenem | | 0.13 | 0.03 | |
| | Amoxicillin | 0.25 | 0.13 | 0.75 |
| Sulopenem | | 0.13 | 0.06 | |
| | Cefuroxime | 0.50 | 0.06 | 0.63 |
| Amoxicillin | | 0.25 | 0.13 | |
| | Cefuroxime | 0.50 | 0.13 | 0.75 |

[a] $MIC_{90}$ is defined as the minimum concentration that inhibits 90% of bacterial growth.

[b] FICI (fractional inhibitory concentration index) was calculated using the concentration at which at least 90% growth inhibition ($MIC_{90}$) of the cultures was observed, as follows: $(MIC_{A\ combi}/MIC_{A\ alone}) + (MIC_{B\ combi}/MIC_{B\ alone})$. A FICI of 0.5 to 1 is defined as additivity.

A checkerboard titration experiment was set up in 96-well microtiter plates, as described previously with minor modifications (1). Increasing concentrations of tebipenem (0.002 to 1 μg/mL), sulopenem (0.002 to 1 μg/mL), cefuroxime (0.02 to 1 μg/mL), and amoxicillin (0.02 to 1 μg/mL) were tested in the presence of 2.5 μg/mL of clavulanate, all dispensed with a Tecan D300e Digital Dispenser. Exponentially grown Mtb cultures ($OD_{600}$ 0.4 to 0.8) were adjusted to a final density of $OD_{600}$ 0.005 in Middlebrook 7H9 broth (BD Difco) supplemented with 10% ADC, 0.2% glycerol and 0.05% Tween 80. Subsequently, 200 μL of this inoculum was added to each well of the microtiter plates. The plates were then sealed with parafilm and incubated for 7 days at 37°C at 110 rpm. After the incubation period, the culture was manually resuspended, and absorbance was measured at $OD_{600}$ using a Tecan Infinite 200 Pro plate reader. To quantify growth inhibition, the initial inoculum density was subtracted from the day 0 measurement, and $MIC_{90}$'s were calculated relative to the untreated control.

**Supplemental Figure 1.**

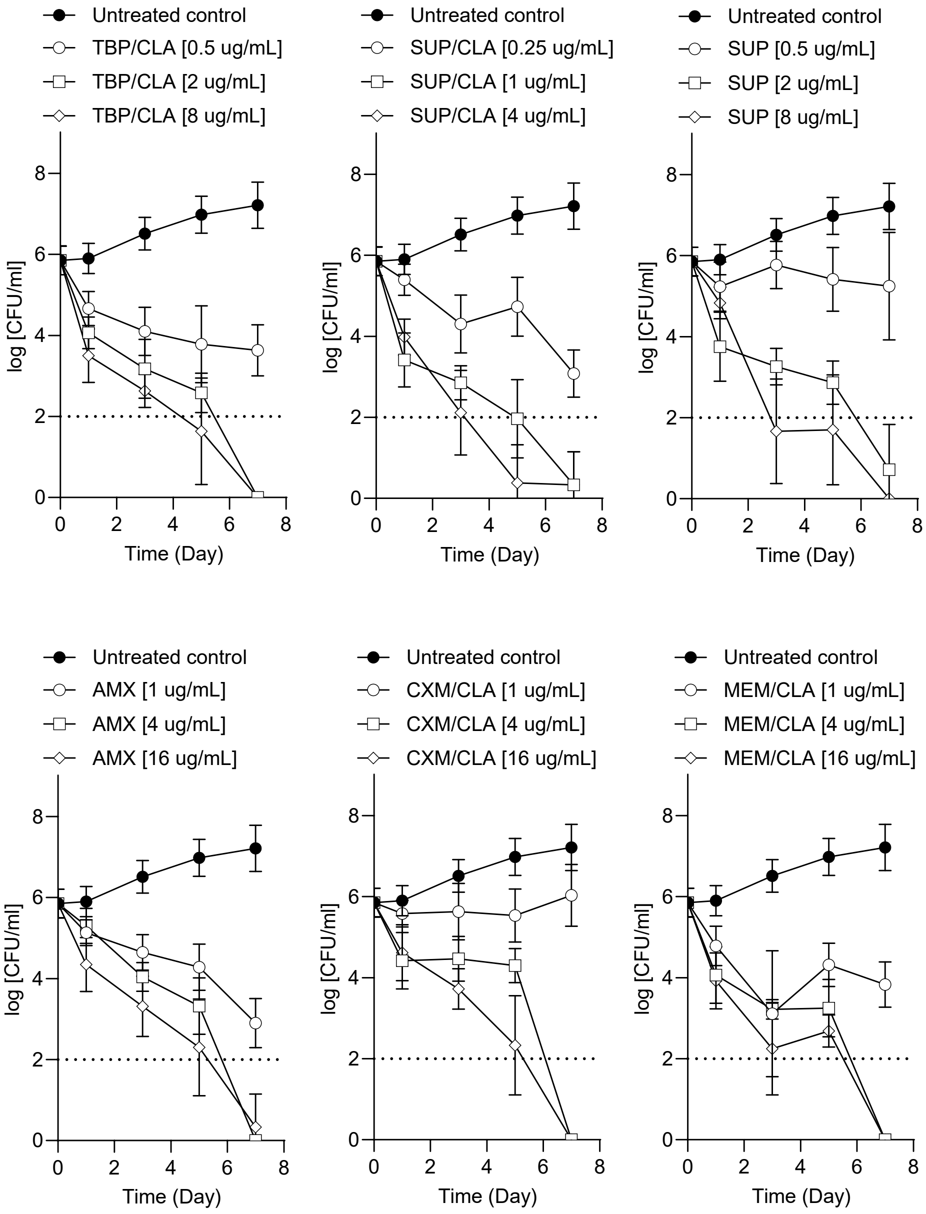


**Figure S1**. Time-kill dose response activity of single agents against Mtb H37Rv. Experiments were performed as described in the main text, at 1x, 4x and 16x the respective MICs in the presence of the β-lactamase inhibitor clavulanate (CLA) (**Table 1**). CLA was added at 2.5 μg/mL when indicated. Meropenem – clavulanate (MEM-CLA) is included as a parenteral control used to treat extensively drug resistant TB patients. Since the growth inhibitory activity of sulopenem (SUP) is largely independent of CLA, we tested SUP and SUP-CLA side by side showing that its bactericidal activity is augmented by CLA. The dotted line indicates the limit of detection or 100 CFU/mL. Experiments were carried out three times independently and mean and SD (error bars) values are shown (GraphPad Prism v10 software).

REFERENCES

1. Aziz DB, Teo JWP, Dartois V, Dick T. 2018. Teicoplanin - Tigecycline Combination Shows Synergy Against Mycobacterium abscessus. Front Microbiol 9:932.
